# Hybrid Path Planning for Unmanned Surface Vehicles in Inland Rivers Based on Collision Avoidance Regulations

**DOI:** 10.3390/s23198326

**Published:** 2023-10-09

**Authors:** Pengcheng Gao, Pengfei Xu, Hongxia Cheng, Xiaoguo Zhou, Daqi Zhu

**Affiliations:** 1 College of Harbor, Coastal and Offshore Engineering, Hohai University, Nanjing 210098, China; hohai_gpc@163.com (P.G.); chenghongxia@hhu.edu.cn (H.C.); 2 School of Naval Architecture and Ocean Engineering, Jiangsu University of Science and Technology, Zhenjiang 212100, China; zhouxg@just.edu.cn; 3 School of Mechanical Engineering, University of Shanghai for Science and Technology, Shanghai 200093, China; zhudaqi@usst.edu.cn

**Keywords:** unmanned surface vehicle in inland rivers, hybrid path planning, collision avoidance regulations in inland rivers, improved A* algorithm, improved model predictive control algorithm

## Abstract

In recent years, with the continuous advancement of the construction of the Yangtze River’s intelligent waterway system, unmanned surface vehicles have been increasingly used in the river’s inland waterways. This article proposes a hybrid path planning method that combines an improved A* algorithm with an improved model predictive control algorithm for the autonomous navigation of the “Jinghai-I” unmanned surface vehicle in inland rivers. To ensure global optimization, the heuristic function was refined in the A* algorithm. Additionally, constraints such as channel boundaries and courses were added to the cost function of A* and the planned path was smoothed to meet the collision avoidance regulations for inland rivers. The model predictive control algorithm incorporated a new path-deviation cost while imposing a cost constraint on the yaw angle, significantly minimizing the path-tracking error. Furthermore, the improved model predictive control algorithm took into account the requirements of rules in the cost function and adopted different collision avoidance parameters for different encounter scenarios, improving the rationality of local path planning. Finally, the proposed algorithm’s effectiveness was verified through simulation experiments that closely approximated real-world navigation conditions.

## 1. Introduction

In recent years, with the continuous development of the Yangtze River’s smart navigation system, intelligent items of equipment, such as unmanned surface vehicles (USVs), have been increasingly used in the inland waterways of the Yangtze River. USVs have become powerful tools for inland riverine environmental surveillance, hydrological assessment, and geomorphic exploration, due to their safety, economic advantages, and other benefits. When operating in inland rivers, the primary challenge for USVs is how to achieve safe and efficient autonomous navigation. Typically, an autonomous navigation system for USVs consists of three modules: perception, path planning, and motion control [1]. As a key technology for USVs to navigate autonomously, effective path planning is crucial in ensuring the safety and efficiency of USVs while they operate in inland waterways. The primary function of path planning is to plan a collision-free path from the starting point to the destination point of a USV, based on environmental information obtained by the USV’s perception module [2]. Depending on the level of environmental information obtained by such perception modules, path planning can be divided into two categories: global path planning and local path planning.

Global path planning algorithms primarily plan a globally optimal path based on a priori information, including known static obstacles, channel boundaries, and water depths. In contrast to local path planning, global path planning commonly suffers from drawbacks such as poor real-time responsiveness and an inability to effectively consider real-time changes in the kinematics of USVs and the dynamic environment of the waterway. Among the widely used global-path planning algorithms are Dijkstra’s algorithm [3], the A* algorithm [4], the rapidly-exploring random tree (RRT) algorithm [5], the particle swarm optimization (PSO) algorithm [6], and neural-network-based algorithms [7]. 

Dijkstra’s algorithm is a depth-first search method that is used to calculate the shortest path between a node and other nodes. Although simple and clear, Dijkstra’s algorithm has low computational efficiency and is not suitable for path planning in large scenes. The RRT algorithm replaces the directed-graph structure with a tree structure, avoiding spatial modeling. However, like Dijkstra’s algorithm, the RRT algorithm has poor real-time performance due to its uniform search characteristics, and it is difficult to obtain global optimal solutions with the RRT algorithm, due to its random-sampling nature. The PSO algorithm is an evolutionary algorithm inspired by the foraging behavior of birds. Particle swarm optimization has the advantages of fast search speed and memory, but it may be limited to local optimization due to premature convergence. Neural-network-based methods have become hot research topics in recent years, but due to their complex computations and other issues, they remain problematic for practical applications.

Compared to the above algorithms, the A* algorithm has a simple structure and operates faster than Dijkstra’s algorithm. The A* algorithm is widely used in the global path planning of USVs, due to its ability to find the globally optimal path—if such a path exists. Ding et al. [8] utilized a complete-coverage neural-network algorithm for the global path planning of USVs vehicles and improved the A* algorithm to help the traversal algorithm escape deadlocks. Simulation experiments validated the effectiveness of their proposed algorithm. Shen et al. [9] aimed to enhance the collision avoidance capability of USVs in open waters by utilizing the A* algorithm and combining ship-maneuvering characteristics. By fully considering navigation-experience regulations, they proposed parallel decision-making dynamic collision avoidance algorithms that only change the heading, or simultaneously change the heading and the speed. Simulation experiments and direct ship-model experiments validated the effectiveness of their proposed algorithms.

Local path planning algorithms, which are also known as local collision avoidance algorithms, are used to generate an optimal or sub-optimal local path for a USV based on real-time information obtained by its onboard sensors, such as water flow, the presence of other ships, and the presence of islands. Compared to global path planning algorithms, local path planning algorithms offer better real-time responsiveness and typically take into account the vessel’s kinematic characteristics and real-time environmental information. However, local path planning algorithms can be susceptible to becoming trapped in local optimal solutions. Common local path planning algorithms include the velocity-obstacle (VO) method [10], the model predictive control (MPC) method [11], the dynamic-window (DW) method [12], and the artificial-potential-field (APF) method [13]. The VO method, the DW method, and the APF method are widely used in local path planning, due to their real-time performances. However, while ensuring real-time performance, these algorithms have limited information considerations and are prone to being limited to local optimal solutions. In addition, the collision avoidance regulations for USVs were difficult to integrate into these algorithms.

Compared to the above algorithms, the MPC algorithm has been increasingly used in the local path planning of USVs due to its receding horizon and its ability to handle multi-input and multi-output nonlinear optimization problems. The MPC algorithm can effectively consider collision avoidance regulations during the optimization process. Johansen et al. [14] employed the MPC algorithm to address the problem of autonomous navigation for USVs open waters. The algorithm considered various factors, such as collision avoidance regulations, water flow, and dynamic obstacles. A large-scale simulation experiment was conducted to validate the effectiveness of the proposed MPC algorithm. Similarly, Eriksen et al. [15] utilized the MPC algorithm for local path planning in open sea environments. This algorithm can consider both moving and static obstacles simultaneously and track a reference trajectory when there is no danger. Once again, simulation experiments were conducted to verify the effectiveness of this algorithm.

A suitable path planning algorithm should aim to achieve a global optimal solution while also accounting for the kinematics of a USV and real-time environmental information. One feasible solution is to combine a global path planning algorithm with a local path planning algorithm, achieving mutual benefit through complementary advantages. This study focuses on the path planning problem of the “Jinghai-I” USV developed by our team for inland rivers. Based on previous research, we propose a hybrid path planning method that combines an improved A* algorithm and an improved model predictive control algorithm. To meet the requirements of riverine navigation regulations, the cost functions for the improved A* algorithm and the improved model predictive control algorithm were modified accordingly. To address the issue of excessive global path turning points, a smoothing method was implemented. In order to enhance the effectiveness of local path planning using model predictive control, different planning parameters were selected, based on different encounter scenarios. The effectiveness of the hybrid path planning method was verified through simulation experiments underwater and by aquatic conditions, both with and without water-flow interference.

The remainder of this paper is organized as follows: Section 1 introduces the research background and its significance; Section 2 describes the basic theory of the USV motion model and hybrid path planning; Section 3 illustrates the simulation results that were conducted; Section 4 discusses the experimental results in detail; and Section 5 summarizes this paper.

## 2. Materials and Methods

### 2.1. Mathematical Model of the USV

**Figure 1:**
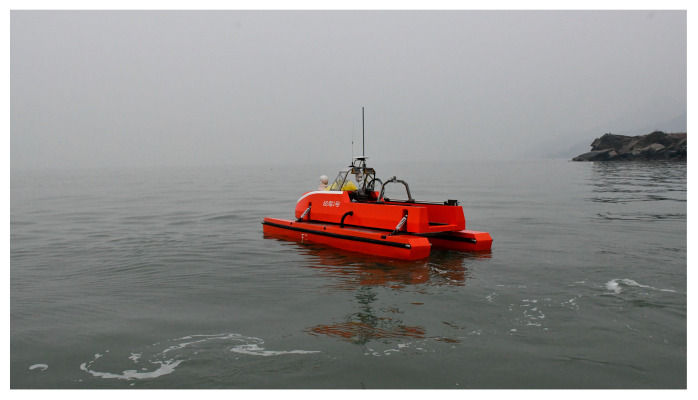
Appearance of “Jinghai-I” USV.

The research and simulation experiments conducted in this paper were based on the “Jinghai-I” USV, which was independently developed by our team. The appearance of the “Jinghai-I” USV is depicted in Figure 1, and its basic parameters are presented in Table 1.

**Figure 2:**
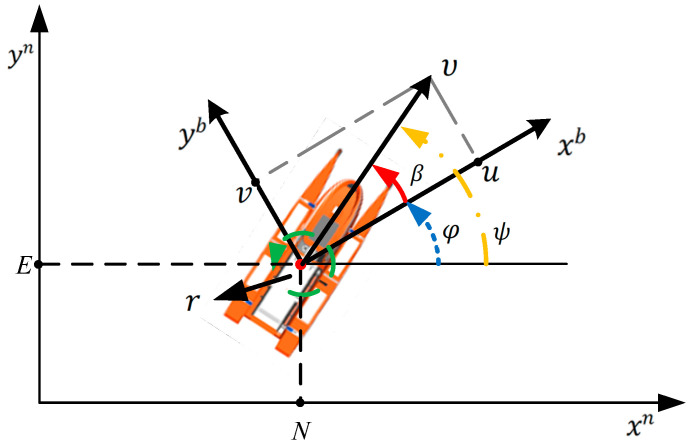
Kinematic model of the 3 DOF “Jinghai-I” USV.

This study utilized a kinematic model of a 3DOF “Jinghai-I” USV, as illustrated in Figure 2, in which $\eta =[x,y,\psi {]}^{T}$ and $v_{current}$ represents the pose of the “Jinghai-I” and the water flow in the earth-fixed reference frame, respectively; $\upsilon =[u,v,r{]}^{T}$ represents the surge speed and the yaw rate of the “Jinghai-I”, respectively; R(ψ) represents the rotation matrix from the body fixed to the earth-fixed frame; ψ=β+φ represents the course of the “Jinghai-I”; β represents the yaw angle (heading) of the “Jinghai-I”; and φ represents the side slip of the “Jinghai-I”. Due to the assumption of zero side slip in this paper, ψ is both the yaw angle (heading) and the course of the “Jinghai-I”. The kinematic equation of the “Jinghai-I” as follows:

(1) $$\dot{\eta }=R(\psi )\nu +v_{current},$$

(2) $$\dot{\psi }=r,$$

(3) $$R(\psi )=\left[\begin{array}{ccc} cos(\psi ) & -sin(\psi ) & 0\\ sin(\psi ) & cos(\psi ) & 0\\ 0 & 0 & 1 \end{array}\right],$$

### 2.2. Hybrid Path Planning of “Jinghai-I” USV

**Figure 3:**
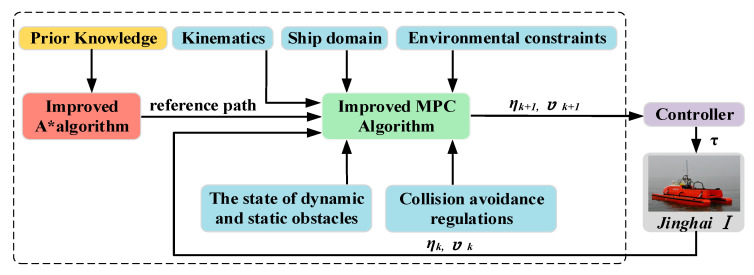
The overall architecture of the hybrid path planning method.

The overall architecture of the proposed hybrid path planning method for the “Jinghai-I” USV is shown in the dashed box in Figure 3. In hybrid path planning, the role of the improved A* algorithm, as the top layer, is mainly to plan a global optimal path for the improved MPC algorithm to follow, in order to avoid the improved MPC algorithm being limited to a local optimum. By tracking the global optimal path, the improved MPC algorithm combined with the kinematics of the model and the parameter information of “Jinghai-I”, the collision avoidance domain, environmental constraints, dynamic and static obstacle states, and collision avoidance regulations in inland rivers to carry out real-time replanning of the path.

#### 2.2.1. Global Path Planning Based on Improved A*

**Figure 4:**
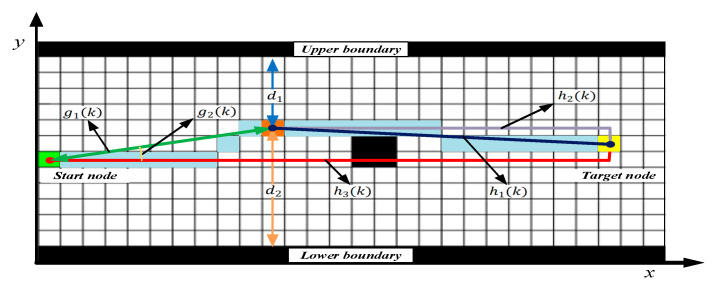
The cost function. The left green box represents the start node and the right yellow box represents the target node.

The improved A* algorithm was used as the top layer of the hybrid path planning approach for “Jinghai-I”. Its main task was to plan a global optimal path from the starting point to the target node, based on the prior knowledge of the environment. As shown in the celestial blue path in Figure 4, this global optimal path not only avoids static obstacles but also guides local path algorithms in tracking the “Jinghai-I”, preventing local path planning algorithms from being limited to local optimal solutions. From a mathematical perspective, the global path planning based on the A* algorithm evaluates the actual cost of node *k* through the cost function *f*(*k*) to minimize the total cost of all nodes in the global path. The form of the optimization problem is as follows:

(4) $$min\sum_{k=1}^{N}f\left(k\right)=g(k)+h(k),$$

where *f*(*k*) represents the actual cost of the optimal path from the starting point to the target point through the current node *k*, and *N* represents the total number of nodes in the path; *g*(*k*) represents the cost value from the current node to the starting node; the heuristic function *h*(*k*) indicates the cost value from the current node to the target node.

In order to meet the maneuverability of the “Jinghai-I” USV and the requirement of inland-navigation regulations, the improved A* algorithm added more cost constraints to the original A* algorithm and adopted a new heuristic function. As a result, the global path optimization and rationality were significantly improved.

##### Design of Evaluation Function

The improved A* algorithm evaluates the cost of the current node k using the following formula:

(5) $$f\left(k\right)=\mu_{1}g_{1}\left(k\right)+\mu_{2}g_{2}\left(k\right)+{\mu_{3}g}_{3}\left(k\right)+h(k),$$

Overall, the cost function $f\left(k\right)$ for the improved A* algorithm consists of four components. Specifically, $\mu_{1}$, $\mu_{2}$ and $\mu_{3}$ are the adjustment parameters of the relevant item, and $g_{1}\left(k\right)$ represents the actual cost from the current node *k* to the start point, which is calculated using the Euclidean distance and expressed as follows:

(6) $$g_{1}\left(k\right)=\sqrt{(x_{cur}-x_{start}{)}^{2}+(y_{cur}-y_{start}{)}^{2}},$$

where ($x_{cur}$, $y_{cur}$) are the coordinates of the current node and ($x_{start}$, $y_{start}$) are the coordinates of the start node.

To ensure the rationality of the planned path, the heading-cost term $g_{2}\left(k\right)$ is added to the g(k), as follows:

(7) $$g_{2}(k)=\left|\arctan\;((y_{cur}-y_{start})/(x_{cur}-x_{start}))\right|/\pi ,$$

Considering the requirements of collision avoidance regulations, the path planned by the improved A* algorithm should avoid obstacles and stay on the right side of the channel as much as possible. Therefore, an additional cost term $g_{3}(k)$ related to the boundary distance is added to g(k), as follows:

(8) $$g_{3}(k)=\left|\frac{\varepsilon_{1}\times d_{1}-\varepsilon_{2}\times d_{2}}{d_{1}+d_{2}}\right|,$$

where $d_{1}$ represents the Euclidean distance from the current node to the upper boundary, and $d_{2}$ represents the Euclidean distance from the current node to the lower boundary. The cost function f(k) components are shown in Figure 4.

In the cost function f(k), h(k) represents the estimated cost from the current node k to the target node after ignoring static obstacles. The rationality of the path planned by the A* algorithm largely depends on the design of the heuristic function. An effective heuristic function not only provides a reasonable estimated cost but also reduces the search space and improves search efficiency. The common methods used to optimize the heuristic function of the A* algorithm include the Manhattan distance method, the Euclidean distance method, the Chebyshev distance method, and dynamic-adjustment heuristic methods. The Manhattan distance method can be directly applied to many grid-based problems, such as chess games. The Chebyshev distance method can be seen as an optimization of the Manhattan distance method, allowing the algorithm to consider diagonal directions when planning. The Euclidean distance method is more suitable for continuous space-planning problems. The A* algorithm, using these three methods as heuristic functions, may sometimes fail to find the optimal solution, while a dynamic adjustment of the heuristic function can provide a more reasonable estimate of the solution.

The form of the dynamic-adjustment heuristic method is as follows:

(9) $$h(k)=h_{1}(k)\times (1+\mu_{4}\times (h_{2}(k)/h_{3}(k))),$$

where $h_{1}(k)$ is the Euclidean distance between the current node and the target node, $h_{2}(k)$ is the Manhattan distance between the current node to the target node, $h_{3}(k)$ represents the Manhattan distance from the starting point to the target node, and $\mu_{4}$ is a coefficient used to adjust the heuristic function.

**Figure 5:**
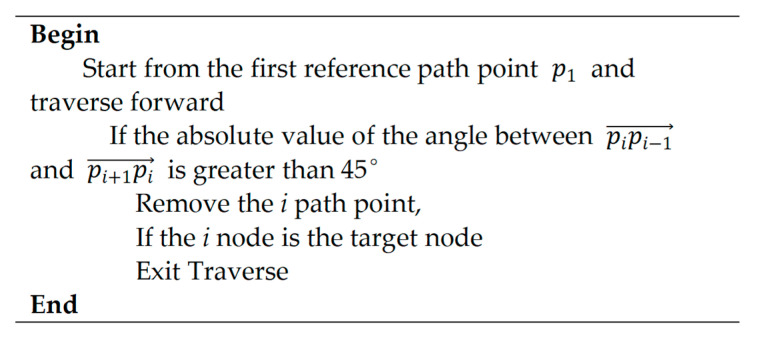
The process of curve smoothing.

**Figure 6:**
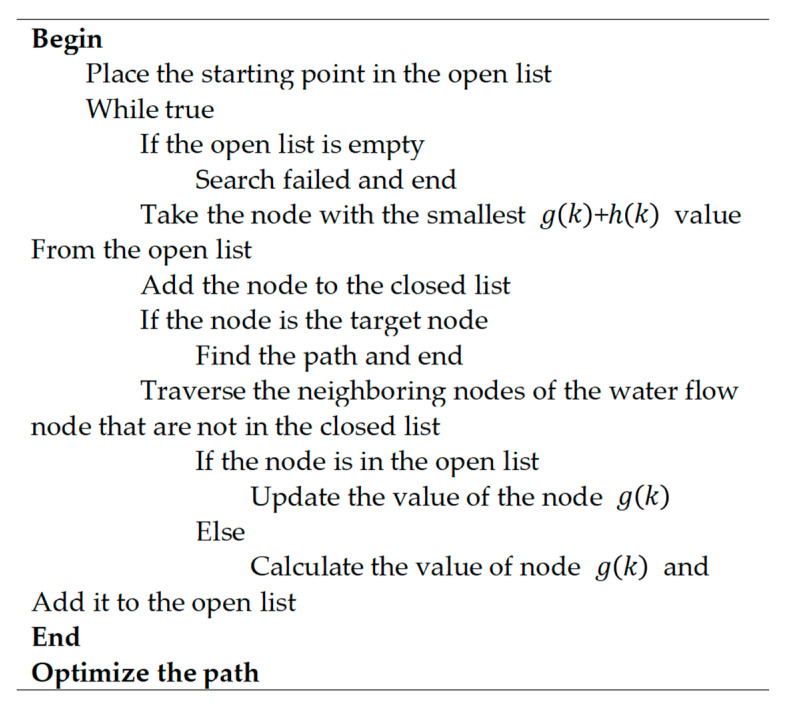
The flow chart of the improved A* algorithm.

In hybrid path planning methods, the tracking performance of local path planning largely depends on the rationality of the global path. The A* algorithm is a global path planning algorithm that is based on grid network. In this study, the planned path has a problem of a too-large turning angle at the corners, which is not convenient for local path planning algorithms to track. Considering the maximum turning-angle limit for a USV in inland river navigation, the planned path by the improved A* algorithm was smoothed. The whole smoothing process is shown in Figure 5 and the flow chart of the improved A* algorithm is shown in Figure 6.

#### 2.2.2. Local Path Planning Based on the Model Predictive Control Algorithm

The model predictive control algorithm is a numerical-optimization algorithm that can effectively handle nonlinear optimization problems with multiple inputs and outputs, It has good robustness with respect to environmental disturbances. Based on previous research [16], this section discusses the optimization of the MPC algorithm according to the actual needs of the project.

##### Collision Avoidance Regulations for Inland Rivers

When navigating in inland rivers, the “Jinghai-I” USV must comply with the requirements of the “Regulation of the People’s Republic of China on Inland River Collision Avoidance”. Unlike the “International Regulations for Preventing Collisions at Sea”, the “Regulation of the People’s Republic of China on Inland River Collision Avoidance” provide more specific provisions on the direction, maximum speed, and maximum yaw angle of a ship to avoid collision during an encounter. The main guidelines for the safe operation of USVs are Articles 9, 10, 11, and 12 of the regulations, which are as follows:

Article 9: Any actions to prevent a collision shall be taken effectively, early, and with good driving skill until the vehicle has passed.

Article 10: Heading.

Two vessels shall come close to meet on the port side of each other except in special circumstances.

Article 11: Overtaking.

When a motorized vessel catches up or overtakes another motorized vessel in a direction greater than 22.5° behind its sway direction, which may pose a danger of collision, it shall be deemed overtaking.

Article 12: Crossing.

When two crossing vessels meet in the same direction of the water flow, if the other vessel is on the starboard side of the vessel, it shall give way to the other vessel.

##### Division of Encounter Situation

**Figure 7:**
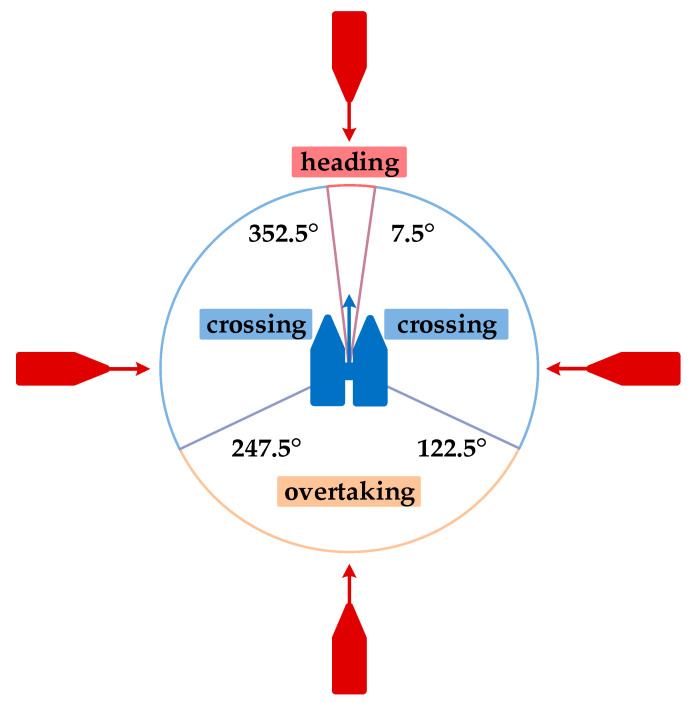
The main encounter scenarios of the “Jinghai-I” USV.

**Figure 8:**
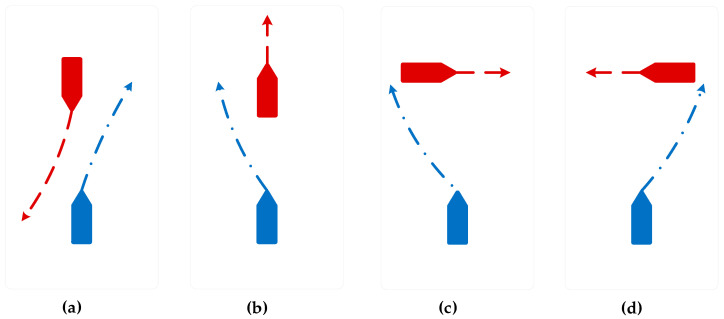
Avoidance and maneuvering of the USV in accordance with regulations. The blue ship is the USV and the red ship is an obstacle ship. Where (**a**) represents heading, (**b**) represents overtaking, (**c**) represents crossing from the port side, and (**d**) represents crossing from the starboard side.

The main encounter situations of USVs in inland rivers are shown in Figure 7, which can be divided into three categories: heading encounters, crossing encounters, and overtaking encounters. According to the requirements of collision avoidance regulations for the “Jinghai-I” USV in inland rivers, the maneuvering for avoiding collisions in the three main encounter situations is shown in Figure 8, where (a) represents heading, (b) represents overtaking, (c) represents crossing from the port side, and (d) represents crossing from the starboard side.

**Figure 9:**
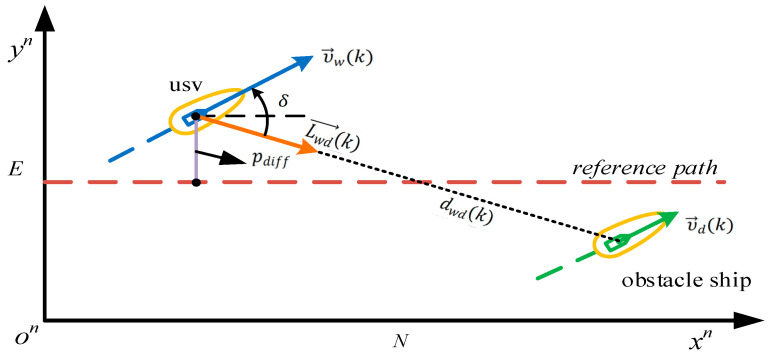
Mathematical model of “Jinghai-I” USV encounter situation.

The mathematical model of encounter situations for the “Jinghai-I” USV is shown in Figure 9. In order to reasonably characterize the safe domain for the “Jinghai-I” USV during river navigation, this paper adopted an improved bumper model to model the safe domain for the “Jinghai-I” USV and obstacle ships [9]. When the surge speed of the “Jinghai-I” USV or the obstacle ship is zero, the size of the safe domain is a circular area with a radius. When the surge speed of the “Jinghai-I” USV or theobstacle ship reaches its maximum, the long axis a=5.2l and the short axis b=0.8l of the ellipse in the front half of the safety domain, where l is the length of the ship [17]. When the speed of the “Jinghai-I” USV or the obstacle ship is the median value, the safety domain has $a=0.8l+4.4l(u/u_{max})$ and b=0.8l, where u is the actual surge speed of the “Jinghai-I” USV and $u_{max}$ is the maximum surge speed of the “Jinghai-I” USV. 

The “Jinghai-I” USV is considered to form a heading encounter with the obstacle ship [14] if $d_{wd}(k)\leq d_{close}$, ${\vec{\upsilon }}_{d}(k)$ is not close to zero, and

(10) $${\vec{\upsilon }}_{w}\left(k\right)\cdot {\vec{\upsilon }}_{d}\left(k\right)<-cos(\delta_{1})\left|{\vec{\upsilon }}_{w}(k)\right|\left|{\vec{\upsilon }}_{d}(k)\right|,$$

(11) $${\vec{\upsilon }}_{w}\left(k\right)\cdot \vec{L_{wd}}\left(k\right)>cos(\delta_{2})\left|{\vec{\upsilon }}_{w}(k)\right|,$$

where $\delta_{1}$, $\delta_{2}$ are angles that meet the requirements of collision avoidance regulations. $d_{wd}(k)$ represents the Euclidean distance between the “Jinghai-I” USV and the obstacle ship at time *k*, $d_{close}$ represents the minimum safe distance for the “Jinghai-I” USV, ${\vec{\upsilon }}_{w}\left(k\right)$ represents the surge-speed vector of the “Jinghai-I” USV at time *k*, ${\vec{\upsilon }}_{d}\left(k\right)$ represents the surge-speed vector of the obstacle ship at time *k*, and $\vec{L_{wd}}\left(k\right)$ represents the direction vector from the “Jinghai-I” USV at time *k* to the obstacle ship.

The “Jinghai-I” USV is considered to be overtaking the obstacle ship if it is behind the obstacle ship, $d_{wd}(k)\leq d_{close}$, and $\left|{\vec{\upsilon }}_{w}(k)\right|>\left|{\vec{\upsilon }}_{d}(k)\right|$, and

(12) $${\vec{\upsilon }}_{w}(k)\cdot {\vec{\upsilon }}_{d}(k)>cos(\delta_{3})\left|{\vec{\upsilon }}_{w}(k)\right|\left|{\vec{\upsilon }}_{d}(k)\right|,$$

where $\delta_{3}$ is the angle that meet the requirements of collision avoidance regulations.

The “Jinghai-I” USV is considered to be crossing the obstacle ship if it faces the obstacle ship, $d_{wd}(k)\leq d_{close}$, and

(13) $${\vec{\upsilon }}_{w}(k)\cdot {\vec{\upsilon }}_{d}(k)<cos(\delta_{4})\left|{\vec{\upsilon }}_{w}(k)\right|\left|{\vec{\upsilon }}_{d}(k)\right|,$$

where $\delta_{4}$ is the angle that meet the requirements of collision avoidance regulations.

Figure 9 provides a schematic representation of the values in Equations (10)–(13). Table 2 shows the actual values of δ for each case. During the specific algorithm-design process, the above expressions were adjusted accordingly, based on the actual collision avoidance requirements.

##### Design of Optimization Problem

The problem of autonomous collision avoidance for USVs is essentially a nonlinear optimization problem (NLP). In this study, the multiple shooting method was adopted to transform the problem of autonomous collision avoidance for the “Jinghai-I” USV into an NLP, and then an ipopt solver based on the Casadi framework [18] was used for solution. In [18], the authors used an MPC algorithm based on the Casadi framework to solve the motion-control problem of mobile robots, and rigorously proved the asymptotic stability of this MPC algorithm. The form of the nonlinear optimization problem is as follows:

(14) $$\underset{\omega }{\min}\emptyset (\omega )\;s.t.g(\omega )=0,\;h(\omega )\leq 0.$$

where ω represents the decision variables, mainly including the pose and velocity of the USV; g(ω) represents the equation constraint of kinematics, such as constraints on initial position; h(ω) represents the inequality constraint, mainly including the distance from obstacles, the distance from the boundary of the channel, the pose and speed of USV; and ∅(ω) represents the objective function that needs to be optimized. In this study, ∅(ω) is defined as follows:

(15) $$\emptyset (\omega )=\sum_{k=1}^{N}(\sigma_{1}{p_{diff}}^{2}+\sigma_{2}{\left(\psi_{k}-\psi_{ref}\right)}^{2}+\sigma_{3}{\left(u_{k}-u_{ref}\right)}^{2}+\sigma_{4}{\left(r_{k}-r_{ref}\right)}^{2})+\sigma_{5}(i_{ow\_s\_i}(m_{s\_i}e^{r_{k}}+\;n_{s\_i}e^{{-r}_{k}}))+\sigma_{6}\emptyset_{rules}+\sum_{k=2}^{N}\left(\sigma_{7}{\left(u_{k}-u_{k-1}\right)}^{2}+\sigma_{8}{\left(r_{k}-r_{k-1}\right)}^{2}\right),$$

where *N* represents the prediction step size of model predictive control; $\sigma_{1}$, $\sigma_{2}$, $\sigma_{3}$, $\sigma_{3}$, $\sigma_{4}$, $\sigma_{5}$, $\sigma_{6}$, $\sigma_{7}$, and $\sigma_{8}$ are tuning parameters of the relevant item; and $p_{diff}$ represents the deviation between the actual position and the reference position of the USV (the details can be seen in Figure 9); $\psi_{k}$ represents the actual heading of the USV at time *k*; $\psi_{ref}$ represents the reference heading of the USV; $u_{k}$ represents the actual surge speed of the USV at time *k*; $u_{ref}$ represents the reference surge speed of the USV; $r_{k}$ represents the actual yaw rate of the USV; $r_{ref}$ represents the reference yaw rate of the USV; $i_{ow\_s\_i}$, $m_{s\_i}$, $n_{s\_i}$ are tuning parameters with a value of 0 or 1; and $\emptyset_{rules}$ represents the cost function related to collision avoidance regulations, as follows:

(16) $$\emptyset_{rules}=\sum_{k=1}^{N}\left(\sigma_{heading}(m_{heading}e^{r_{k}}+n_{heading}e^{{-r}_{k}})+\sigma_{crossing}(m_{crossing}e^{r_{k}}+n_{crossing}e^{{-r}_{k}})+\;\sigma_{overtaking}(m_{overtaking}e^{r_{k}}+n_{overtaking}e^{{-r}_{k}})\right),$$

where $\sigma_{heading}$, $\sigma_{crossing}$, and $\sigma_{overtaking}$ are tuning parameters of the relevant item; $m_{heading}$, $n_{heading}$, $m_{crossing}$, $n_{heading}$, $m_{overtaking}$, and $n_{heading}$ are tuning parameters with a value of either 0 or 1, as illustrated in Section 4.

In the nonlinear programming problem, h(ω) mainly includes the distance constraint between the USV and the obstacle $h_{1}(\omega )$ and the speed increment constraint of the USV, and $h_{1}(\omega )$ consists of static obstacles $h_{1}\left(L_{si}\right)$ and dynamic ships $h_{1}\left(L_{di}\right)$. The specific forms of $h_{1}\left(L_{si}\right)$ are as follows:

(17) $$h_{1}\left(L_{si}\left(t_{1:N+1}\right)\right)=\;\left[\begin{array}{c} \begin{array}{c} {(R}_{si}+{dom}_{ow}(t_{1}))-{\left\|p_{ow}(t_{1})-p_{si}\right\|}_{2}\\ {(R}_{si}+{dom}_{ow}(t_{2}))-{\left\|p_{ow}(t_{2})-p_{si}\right\|}_{2} \end{array}\\ \vdots \\ {(R}_{si}+{dom}_{ow}(t_{N+1}))-{\left\|p_{ow}(t_{N+1})-p_{si}\right\|}_{2} \end{array}\right]\leq 0,$$

where $R_{si}$ represents the radius of the safety domain of static obstacle *i*; ${dom}_{ow}(t_{1:N+1})$ represents the value of the collision avoidance domain of the USV in the direction of static obstacle *i* at time $t_{1:N+1}$; $p_{ow}(t_{1:N+1})$ represents the position of the USV at time $t_{1:N+1}$; and $P_{si}$ represents the position of the static obstacle *i*. 

The specific form of $h_{1}\left(L_{di}\right)$ is as follows:

(18) $$h_{1}\left(L_{di}\left(t_{1:N+1}\right)\right)=\;\left[\begin{array}{c} \begin{array}{c} {(dom}_{di}(t_{1})+{dom}_{ow}(t_{1}))-{\left\|p_{ow}(t_{1})-p_{di}(t_{1})\right\|}_{2}\\ {(dom}_{di}(t_{2})+{dom}_{ow}(t_{2}))-{\left\|p_{ow}(t_{2})-p_{di}(t_{2})\right\|}_{2} \end{array}\\ \vdots \\ {(dom}_{di}(t_{i})+{dom}_{ow}(t_{N+1}))-{\left\|p_{ow}(t_{N+1})-p_{di}(t_{N+1})\right\|}_{2} \end{array}\right]\leq 0,$$

where ${dom}_{di}(t_{1:N+1})$ is the value of the collision avoidance domain in the direction of the USV at time $t_{1:N+1}$ for the dynamic ship *i*; $p_{di}(t_{1:N+1})$ is the location of dynamic ship *i* at time. The specific form of $h_{2}(\omega )$ is as follows:

(19) $$h_{2}\left(u_{1:N}\right)=\left[\begin{array}{c} u_{1:N}-u_{max}\\ u_{min}-u_{1:N} \end{array}\right]\leq 0,$$

(20) $$h_{2}\left(r_{1:N}\right)=\left[\begin{array}{c} r_{1:N}-r_{max}\\ r_{min}-r_{1:N} \end{array}\right]\leq 0,$$

(21) $$h_{2}\left(\left|∆u_{2:N}\right|\right)=\left[\left|{∆u}_{2:N}\right|-∆u_{max}\right]\leq 0,$$

(22) $$h_{2}\left(\left|∆r_{2:N}\right|\right)=\left[\left|{∆r}_{2:N}\right|-∆r_{max}\right]\leq 0,$$

where $u_{1:N}$ and $r_{1:N}$ represent the surge speed and the yaw rate of the USV at the prediction step, respectively; ${∆u}_{2:N}$ and ${∆r}_{2:N}$ represent the increments of the surge speed and the yaw rate of the USV at the predicted step, respectively; $u_{max}$, $u_{min}$ represent the maximum and minimum surge speed of the USV, respectively; $r_{max}$, $r_{min}$ represent the maximum and minimum yaw rates of the USV, respectively; and ${∆u}_{max}$, ${∆r}_{max}$ represent the maximum surge speed and yaw rate increment of the USV, respectively.

In addition, due to the limitations of inland-navigation conditions, a USV usually adopts a steering angle of no greater than 40° when making evasive maneuvers. Therefore, the constraint of the maximum heading angle is also taken into account in the nonlinear programming problem.

## 3. Results

The purpose of this simulation study was to verify the performance of the proposed hybrid path planning method. A typical scenario for the “Jinghai-I” USV in inland waterways was considered in the simulation. The simulation results were illustrated by graphs representing situation snapshots.

### 3.1. Global Path Planning Based on Improved A* Algorithm

#### 3.1.1. Simulation Settings

In the simulation, it was assumed that the “Jinghai-I” USV was navigating in an inland waterway of 550×220 square meters, and the information of two static obstacles was known. Since the length of the “Jinghai-I” USV is 5 meters, the grid size was set to 25 square meters. The simulation conditions were carried out in MATLAB R2020a on a 1.6 GHz Intel Core i5 operating system. The detailed parameters of the simulation are shown in Table 3.

In the simulation results, the following symbols and colors were used:

- The green square represents the starting node and the yellow square represents the target node;
- The black area indicates the “Jinghai-I” USV’s inaccessible zones, including two static obstacles and the boundary of the channel;
- The white area indicates the “Jinghai-I” USV’s accessible zones.

#### 3.1.2. Simulation Results

##### Different Heuristic Functions h(k)

In order to compare the advantages and disadvantages of different heuristic functions, we conducted simulation experiments on the A* algorithm after adopting different heuristic functions. In these simulation results, the following symbols and colors were used:

- The red circle line is the global optimal path planned by the improved A* algorithm;
- The yellow hexagon star represents the optimal path planned by the A* algorithm using the Manhattan method as the heuristic function;
- The green square represents the optimal path planned by the A* algorithm using the octave distance method as the heuristic function;
- The orange triangle represents the optimal path planned by the A* algorithm using the Euclidean method as the heuristic function.

**Figure 10:**
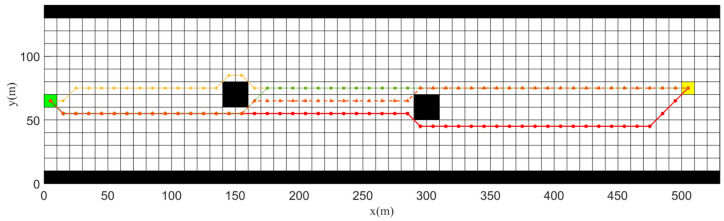
Simulation results of different heuristic functions h(k).

The simulation results, as shown in Figure 10, indicate that the path planned by using the dynamic-adjustment heuristic method better met the design requirements of the algorithm. The global path planned by using the dynamic-adjustment heuristic method entirely avoided obstacles, while being closer to the right side of the channel with fewer turning points. The A* algorithm planned using the Manhattan distance as the heuristic function had the most turning points among all paths. Although the A* algorithm planned using the Octave distance as the heuristic function had the same number of turning points as the dynamic-adjustment heuristic function, it planned a path that was closer to the middle of the channel and did not meet the original intention of the algorithm design. The average time required for each heuristic function to run five times is provided in Table 4. It can be seen that the dynamic-adjustment heuristic function took longer to plan because it considered more factors, but its time was still significantly shorter than the time required by the Euclidean distance method.

##### Different Coefficients $\mu_{4}$

$\mu_{4}$ is a parameter used to adjust the dynamic-adjustment heuristic function, and different values of $\mu_{4}$ lead to different results in the planning of the improved A* algorithm. In order to study the influence of $\mu_{4}$ clearly, we compared the effects of the improved A* algorithm planned under different values of $\mu_{4}$.

In these simulation results, the following symbols and colors were used:

- The blue lower triangle shows the global optimal path planned by the improved A* algorithm when $\mu_{4}$ = 0.02;
- The red dot triangle represents the global optimal path planned by the improved A* algorithm when $\mu_{4}$ = 0.045;
- The blue upper triangle represents the global optimal path planned by the improved A* algorithm when $\mu_{4}$ = 0.6;
- The orange pentagram represents the global optimal path planned by the improved A* algorithm when $\mu_{4}$ = 6;
- The golden diamond represents the global optimal path planned by the improved A* algorithm when $\mu_{4}$ = 60;
- The green square represents the global optimal path planned by the improved A* algorithm when $\mu_{4}$ = 600;
- The sky-blue hexagram is the globally optimal path planned by the improved A* algorithm when $\mu_{4}$ = 6000.

**Figure 11:**
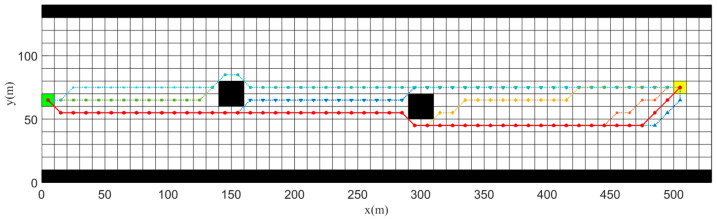
Simulation results for different $\mu_{4}$.

The simulation results shown in Figure 11 indicate that when $\mu_{4}$ is too small, the path planned by using the dynamic-adjustment heuristic function overlaps with Euclidean distance method. When $\mu_{4}$ is too large, the path planned has a slightly larger turning point and coincides with the Manhattan distance method. After experiments, it was found that the path planned when $\mu_{4}\in [0.036 0.057]$ was reasonable, with a closer proximity to the right side of the channel and the lowest number of turning points. Therefore, the final value of $\mu_{4}$ in this study was 0.045.

##### Path Smoothing

**Figure 12:**
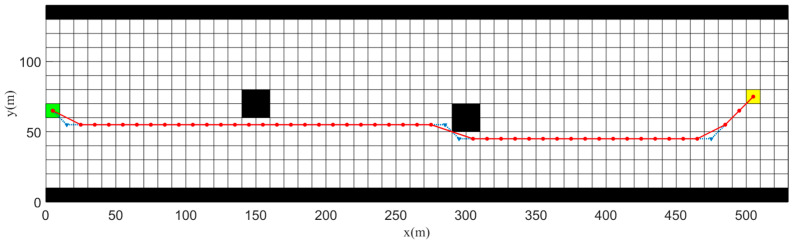
Simulation results before and after path smoothing. The red circular dots represent the smoothed path, while the blue lower triangle represents the original path.

As the A* algorithm is a global path planning algorithm based on a grid network, under the grid size of 5×5 m used in this study there will be problems with excessive turning angles in the planned global path. This not only fails to meet the requirements of maximum heading angle set by river-navigation regulations, but also makes it difficult for local path planning algorithms to track. To address this issue, the paths planned by the A* algorithm were smoothed, as shown in Figure 12. Based on the simulation results, it can be seen that the points in the original path may have a turning angle at the turning point that exceeds the maximum angle set by river-navigation regulations, while the maximum turning angle after smoothing was 20 degrees. This ensured that the planned path met the requirements of river-navigation regulations and made it easier for local path planning algorithms to follow.

### 3.2. Local Path Planning Based on the MPC Algorithm

The local path planning algorithm based on the MPC algorithm [16] was improved according to the actual requirements of the project. To verify the performance of the improved algorithm, three simulation results using three cost functions were carried out under the conditions of water flow and no water flow. The cost function *I* removed the distance constraint of obstacles and used different collision avoidance parameters for different encounter scenarios, which was an improvement over the previous study. The cost function *II* added the heading constraint ${\left(\chi_{k}-\chi_{ref}\right)}^{2}$ to the cost function *I*. The cost function *III* replaced the original position deviation constraint ${\left\|\eta_{k}-\eta_{ref}\right\|}_{2}$ with ${p_{diff}}^{2}$, which was another improvement. Finally, a detailed comparative study of the planning results under typical scenarios was conducted for the three cost functions.

#### 3.2.1. Simulation Settings

In this part of the simulation, we assumed that the water flow was ideal and the specific information of the obstacle ship could be obtained through real-time AIS on board. The simulation conditions were consistent with those stated in Section 3.1.1. In the simulation, the fixed parameters used by the MPC algorithm were those that are stated in Table 5, while the parameters that adapted to different encounter scenarios were those that are stated in Table 6.

In these simulation results, the following symbols and colors were used:

- The yellow boat represents the “Jinghai-I” USV and the blue boat represents the obstacle boat;
- The golden dynamic area indicates the dynamic bumper domain of the “Jinghai-I” USV and the obstacle ship;
- The purple dotted line represents the reference path of the “Jinghai-I” USV;
- The blue dotted line represents the path planned after the cost function *I*;
- The green dotted line represents the path planned by the cost function *II*;
- The orange solid line represents the path planned by the cost function *III*;
- The dotted line in light blue indicates the navigation path of the obstacle ship.

#### 3.2.2. Simulation Result

##### Local Path planning Results without Water Flow

**Figure 13:**
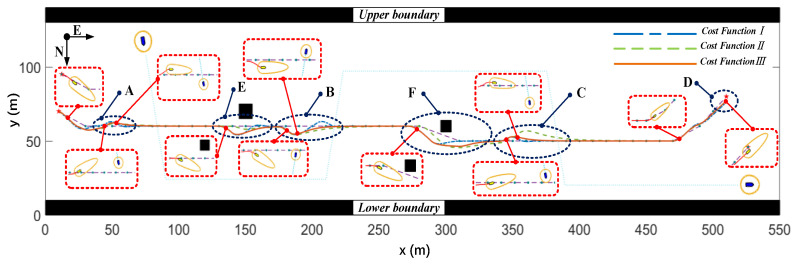
The local optimal paths planned by the three cost functions under the condition of no water flow.

In this section, the local path planning results of the MPC algorithm using three different cost functions are presented under the condition of no water flow. The encounter situations of the “Jinghai-I” USV with cost function *III* applied to typical scenarios are shown in the red dashed boxes in Figure 13. As shown in Figure 13, deviations between the planned paths and the reference paths for A, B, C, and D scenarios were smaller when cost function *III* was used. In the E and F scenarios, the distance between the planned path and static obstacles was larger when cost function *III* was used, which reduced the collision risk of the “Jinghai-I” USV. In scenarios B and C, due to the absence of the heading constraint, the planned path of cost function *I* had an S-shaped trajectory, thus increasing the maneuverability of the USV.

**Figure 14:**
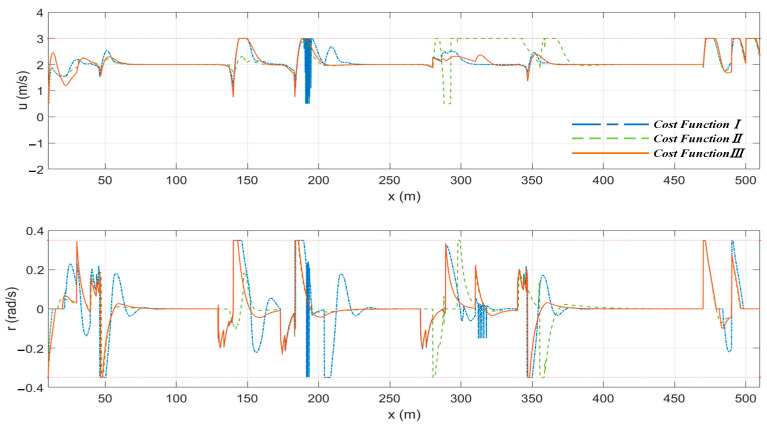
The speed of the “Jinghai-I” USV planned by the three cost functions under the condition of no water flow.

Figure 14 shows the speed of the “Jinghai-I” USV planned by the algorithm under three different cost functions. As shown in Figure 14, there were obvious oscillations in the surge speed and the yaw rate obtained by the MPC algorithm using cost function *I*. The amplitude of changes in the surge speed and the yaw rate obtained by the MPC using cost function *II* was significantly higher than that obtained by using cost function *III*.

Table 7 shows the path deviation and the heading-angle deviation of the MPC algorithm under three different cost functions. It can be observed that after adding the heading-angle-deviation cost, the total errors of the path ${error}_{total\_p}$ for cost functions *II* and *III* were reduced by 7.93% and 8.15%, respectively, compared to cost the error for function *I*. The total heading-angle-deviation errors, ${error}_{total\_r}$, were also reduced by 39.26% and 34.08% for cost functions *II* and *III*, respectively. Although the path deviation of the third cost function was significantly higher than that of cost function *II* under encounter scenarios E and F, the total deviation of cost function *III* was close to that of cost function *II*, indicating that the tracking ability of cost function *III* on the reference path was better than that of cost function *II*.

##### Local Path planning Results with Water Flow

**Figure 15:**
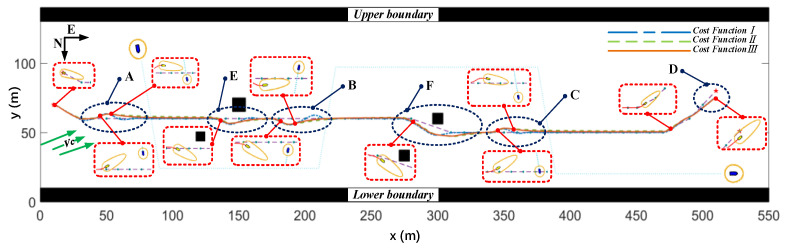
The optimal paths planned by three cost functions under the condition of water flow.

This section provides the path planning results of the MPC algorithm using three different cost functions under the condition of water flow. The encounter situation of “Jinghai-I” USV when using cost function *III* in typical scenarios is shown in the red dashed box in Figure 15, with the green arrow representing the actual direction of water flow in the simulation. Similarly, it can be observed from Figure 15 that the deviations between the planned paths and the reference paths for cost functions *II* and *III* were significantly smaller than those for cost function *I*. Table 7 shows that under the condition of water flow, the total errors of the paths ${error}_{total\_p}$ planned by cost functions *II* and *III* were reduced by 23.53% and 15.33%, respectively, compared to the error of cost function *I*, while the total heading-angle deviation ${error}_{total\_r}$ was reduced by 40.22% and 34.36%, respectively. 

**Figure 16:**
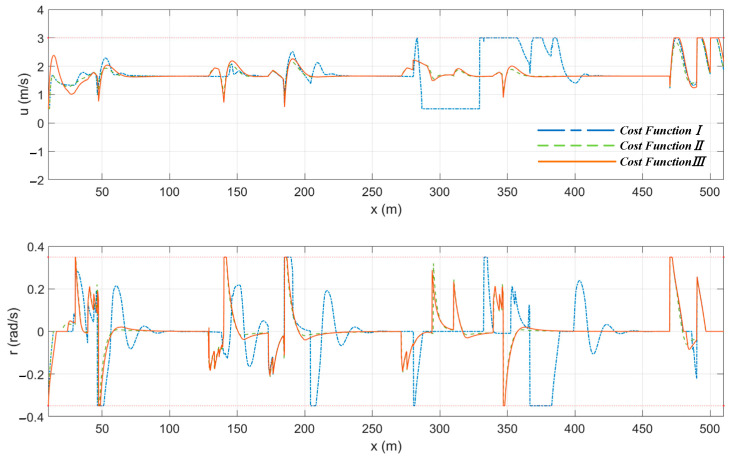
The speed of the “Jinghai-I” USV planned by the three cost functions under the condition of water flow.

As can be seen from the velocity graph shown in Figure 16, cost function *I* still had a large variation in speed. Surprisingly, the velocity variations of cost functions *II* and *III* were almost identical. This was mainly due to the fact that avoidance actions are mostly carried out in the direction of flow resistance. To counteract water-flow interference, the MPC algorithm performed a receding horizon while considering water-flow factors. This also demonstrated that the local path algorithm based on the MPC algorithm had good robustness to water flow. From the overall trajectory graph shown in Figure 15 and the deviations provided in Table 7, it can be concluded that cost function *III* outperformed cost function *II*.

## 4. Discussion

This study proposed a hybrid path planning method that combined the improved A* algorithm and the improved MPC algorithm to solve the problem of path planning for the “Jinghai-I” USV, which was independently developed by the team for autonomous navigation in inland rivers.

To ensure that the planned global path complied with the requirements of inland-navigation regulations for all vessels to travel as far as possible on the right side of the waterway, a cost term based on the distance from the waterway boundary was added to the cost function g(k) of the A* algorithm.

To ensure the global optimization of the path, a dynamic-adjustment heuristic method was used as the heuristic function of the improved A* algorithm, and different values of $\mu_{4}$ were experimented with to determine the appropriate range. In this study, the planned path by the improved A* algorithm had excessive turning angles, which did not meet the requirements of the maximum heading angle in river navigation. Finally, the global optimal path planned by the improved A* algorithm was smoothed. 

When performing path smoothing, conventional curve smoothing methods such as B-spline curves and polynomial fitting were not used. Instead, unreasonable path points were removed to achieve curve smoothing. This was because, based on MPC local path planning, a more locally optimal path that conforms to the kinematics of the “Jinghai-I” USV could be planned. The improved A* algorithm only needed to plan a globally optimal path that met the maximum heading-angle requirements of river navigation, thus guiding the local path planning algorithm for tracking.

On the basis of tracking the global optimal path, the “Jinghai-I” USV used the MPC algorithm for local path re-planning. The MPC algorithm used different parameters according to different encounter situations, thus planning a more reasonable path. In response to the shortcomings of previous research, the MPC algorithm introduced a new path-deviation cost and a new yaw-angle cost, which significantly reduced the error of the algorithm in tracking the reference path and the yaw angle. Simulation experiments validated the effectiveness of the improved model predictive control algorithm.

As indicated in Formula (15), increasing the tuning coefficient increases the proportion of the corresponding cost term in the overall cost function, making the corresponding constraint more effective. However, going too far is as bad as not going far enough. An excessively large $\sigma_{1}$ causes the algorithm to overemphasize the tracking of the reference path, affecting the actual planning efficiency and even leading to collision avoidance failure of the “Jinghai-I” USV. The selection of the reference angle is similar to the LOS method, and an excessively large $\sigma_{2}$ will result in the “Jinghai-I” USV failing to return to the reference trajectory after avoiding obstacles. An excessively large $\sigma_{3}$, $\sigma_{4}$, $\sigma_{7}$, and $\sigma_{8}$ will cause the algorithm to focus more on maintaining speed during planning, reducing the flexibility of collision avoidance. In normal circumstances, the value of $i_{ow\_s\_i}$ is 0. Only when static obstacles are within the range of collision avoidance for the “Jinghai-I” USV will the value of $i_{ow\_s\_i}$ be 1. At this time, the corresponding cost term will come into play and guide the “Jinghai-I” USV to avoid static obstacles. The values of $m_{s\_i}$ and $n_{s\_i}$ are also similar, but they will not both be 1 at the same time. For example, when static obstacle $s_{i}$ enters the collision avoidance range of the “Jinghai-I” USV at time *k*, assume that static obstacle $s_{i}$ is on the left side of the heading direction of the “Jinghai-I” USV and that the heading angle is positive when the “Jinghai-I” USV turns right. At this time, the value of $n_{s\_i}$ is 1 and the value of $m_{s\_i}$ is 0. The algorithm guides the “Jinghai-I” USV to turn right as far as possible to avoid the static obstacle. This maneuver not only reduces the collision risk of the “Jinghai-I” USV but also meets the requirements of inland-navigation regulations. When the static obstacle leaves the collision avoidance range of the “Jinghai-I” USV, the value of $i_{ow\_s\_i}$ is 0, and other cost items will constrain the “Jinghai-I” USV to continue to follow the reference path. Similarly, the values of coefficients in Formula (16) also follow this principle.

After adding the water flow term, the deviation of the planned path by cost function *I* was smaller than that without water-flow interference. This was mainly because the direction of the flow was opposite to the direction that the “Jinghai-I” USV evaded. Under the influence of the flow, the deviation on the right side of the path planned by cost function *I* became smaller. In addition, the gap between cost functions *II* and *III* did not seem as obvious as before, without flow interference, mainly due to the impact of the flow. For example, in scenario C shown in Figure 13, cost function *II* indicated that it was acceptable for there to be a certain path deviation. However, due to the disturbance caused by the water flow, this path deviation became even larger, making its proportion in the cost function higher. To minimize the cost function, the algorithm re-planned a more reasonable path. From another perspective, because of the receding horizon characteristic of the MPC algorithm, it has good robustness against water flow disturbances. Compared with [16], this study removed the distance penalty term from the cost function and considered the distance factor during encounter-situation judgment. This not only did not reduce the performance of the algorithm, but also simplified the cost function and improved the efficiency of the algorithm.

Although various encounter situations were considered in the algorithm, the main focus of the simulation was on crossing encounters, as they posed more challenges for the “Jinghai-I” USV. The speed of obstacle ships also varied during the simulation, but due to the robustness of the MPC algorithm, the “Jinghai-I” USV still effectively avoided obstacles. For the performance of the algorithm in other encounter scenarios, please refer to the authors’ previous study [16].

## 5. Conclusions

In order to address the path planning problem of the “Jinghai-I” USV during autonomous navigation in inland rivers, this study proposed a hybrid path planning method that combines an improved A* algorithm with an improved model predictive control algorithm. The A* algorithm adopted a dynamic adjustment function as the heuristic function to ensure global optimization in the planned path. The cost function of the improved A* algorithm included channel boundary constraints and heading constraints, resulting in a globally optimal path that met both river-navigation regulations and local path-tracking requirements. In response to previous research limitations, the model predictive control algorithm was modified accordingly. The cost function of the MPC algorithm included a new path-deviation cost and an additional heading cost, which significantly reduced the tracking deviation and the heading-angle deviation of the algorithm. For different encounter scenarios, the MPC algorithm adopted different collision avoidance parameters, further improving local path planning performance. Finally, through simulation experiments, the effectiveness of the proposed hybrid path planning method was demonstrated. 

Based on previous research, some improvements were made to the hybrid path planning algorithm in response to practical requirements. However, there is still much work to be done to truly enable the “Jinghai-I” USV to navigate autonomously in rivers. In the future, we propose the following:

- Continuing to study more complex collision avoidance scenarios for the “Jinghai-I” USV in inland rivers, particularly when it encounters obstacles such as other ships, and further optimizing the collision avoidance behavior of the “Jinghai-I” USV in these scenarios;
- Conducting future research to improve the applicability of the current mathematical models used to differentiate between encounter scenarios, as they are not accurate for some of these scenarios; and
- Validating the research in this paper through practical testing on the “Jinghai-I” USV.

## Figures and Tables

**Table 1:** Basic parameters of “Jinghai-I” USV.

Parameter	Value	Parameter	Value
length	5 m	maximum speed	6 kn
wide	2 m	cruise speed	3~4 kn
height	1.5 m	acceleration	$0.3\;m/s^{2}$
full load draft	≤0.5 m	maximum yaw rate	$15\;{}^{\circ}/s^{2}$
full load displacement	≥1150 kg	average yaw rate	$5\sim 10\;{}^{\circ}/s^{2}$

**Table 2:** Values of δ that meet the requirements of collision avoidance regulations.

Parameter	Value
$\delta_{1}$	40°
$\delta_{2}$	7.5°
$\delta_{3}$	68.5°
$\delta_{4}$	68.5°

**Table 3:** Detailed parameters of the A* algorithm.

Parameter	Value	Parameter	Value
The size of the grid	5 × 5 m	$\mu_{3}$	10
$\mu_{1}$	0.1	$\varepsilon_{1}$	0.5
$\mu_{2}$	1	$\varepsilon_{2}$	0.7

**Table 4:** Average running time of different heuristic functions.

h(k)	Average Time/s
Manhattan distance method	0.0786
Octave distance method	0.0812
Euclidean distance method	0.2239
Dynamic-adjustment heuristic method	0.1552

**Table 5:** Fixed parameters of the improved MPC algorithm.

Parameter	Value	Parameter	Value
*L*	5 m	$\left[u_{min},u_{max}\right]$	[0.5,3] m/s
*N*	20	$\left[r_{min},r_{max}\right]$	$\left[-10,10\right]\;{}^{\circ}/s$
*T*	0.1 s	$\left[{\dot{u}}_{min},{\dot{u}}_{max}\right]$	$\left[-0.05,0.3\right]\;m/s^{2}$
$d_{close\_d\_min}$	25 m	$\left[{\dot{r}}_{min},{\dot{r}}_{max}\right]$	$[-0.6,0.6]\;{}^{\circ}/s^{2}$
$d_{close\_s\_min}$	15 m	$\sigma_{4}$	0.2
$d_{close\_s\_pre}$	20 m	$\sigma_{5}$	10
$d_{close\_d\_pre}$	30 m	$\sigma_{6}$	10
$|\chi {|}_{max}$	(2π/9) rad	$\sigma_{7}$	0.5
$u_{ref}$	2 m/s	$\sigma_{8}$	0.5

**Table 6:** Adapting parameters of the improved MPC algorithm to different scenarios.

Encounter Scenarios	$\sigma_{1}$	$\sigma_{2}$	$\sigma_{3}$	$\sigma_{heading}$	$\sigma_{crossing}$	$\sigma_{overtaking}$	$i_{ow\_s\_i}$
No obstacles	2.5	1.1	7	0	0	0	0
Crossing	0.7	0.2	7	0	1	0	0
Overtaking	0.5	0.1	5	0	0	1	0
Heading	0.7	0.2	5	1	0	0	0
Only static obstacles	0.25	0.1	7	0	0	0	1

**Table 7:** Path and yaw angle errors of three cost functions. Here, ${error}_{max\_A}$ represents the maximum path deviation for Region A.

Cost Function	$v_{c}$	${error}_{total\_p}$	${error}_{max\_A}$	${error}_{max\_B}$	${error}_{max\_C}$	${error}_{max\_D}$	${error}_{total\_r}$
I	×	99.0318 m	2.9882 m	5.7263 m	2.6925 m	3.9568 m	17.3502 rad
II	×	91.1743 m	1.8611 m	5.9423 m	6.8505 m	3.0001 m	10.5379 rad
III	×	90.9654 m	2.1878 m	5.3729 m	2.6234 m	2.8795 m	11.4376 rad
I	√	93.4238 m	3.4382 m	4.5093 m	2.5324 m	4.3987 m	16.7966 rad
II	√	77.4369 m	3.2110 m	4.3026 m	2.8485 m	4.8261 m	10.0409 rad
III	√	79.1045 m	3.2743 m	4.0516 m	3.0425 m	4.5786 m	11.0247 rad

## Data Availability

All data, together with relevant analysis scripts and files, are available from the corresponding author upon request.
